# Self-Perception of Gerontoism according to Social Support and Family Functionality

**Published:** 2019-04

**Authors:** Herbert Rubens KOCH FILHO, Luiza Foltran de Azevedo KOCH, Solena Ziemer KUSMA, Sérgio Aparecido IGNÁCIO, Simone Tetu MOYSÉS, Luciana Ribeiro Azevedo ALANIS, Marilisa Carneiro Leão GABARDO, Samuel Jorge MOYSÉS

**Affiliations:** 1. School of Life Sciences, Pontifícia Universidade Católica do Paraná, Rua Imaculada Conceição 1155, 80215-901, Curitiba, Paraná, Brazil; 2. School of Health Sciences, Rua Professor Pedro Viriato Parigot de Souza 5300, 81280-330, Curitiba, Paraná, Brazil; 3. School of Medicine, Pontifícia Universidade Católica do Paraná, Rua Imaculada Conceição 1155, 80215-901, Curitiba, Paraná, Brazil

**Keywords:** Elderly, Family functionality, Prejudice, Social support

## Abstract

**Background:**

The gerontoism, a neologism adopted here, is a form of discrimination regarding age and can occur in rational, emotional, and behavioral contexts, and could be influenced by many factors. This study aimed to identify if the social support and the family functionality interfere in the self-perception of gerontoism.

**Methods:**

Participants were 376 elderly in good physical and mental condition. They participated in the Group Living organization in the Municipality of Curitiba, State of Paraná, Brazil, in 2012. Information was collected about sociodemographic profile using a structured questionnaire. The social support, the family functionality, and the self-perception of gerontoism were defined by the Medical Outcomes Study, the Family APGAR index, and Ageism Survey, respectively. The variables were analyzed by Pearson’s correlation coefficient, One Way ANOVA, Tukey’s HSD test, and the Student’s t-test.

**Results:**

Most of the participants came from small cities from the interior (48.7%), were female (94.4%), with age ranging 60–69 yr-old (45.5%), whites (76.1%), widowers (47.1%), with children (93.9%), with low schooling (55.3%), with family income from 1–2 minimum wage (31.4%), and retirees not working (44.1%). It was not observed correlation between sociodemographic variables and self-perception of gerontoism. Statistical significance was observed between self-perception of gerontoism and social support (*r*= −0.26, *P*=0.00), and between the self-perception and family functionality (*r* = −0.28, *P*=0.00). Once the scores of self-perception of gerontoism increased, the ones from social support and family functionality, decreased.

**Conclusion:**

Lower self-perception of gerontoism was observed in elderly with higher social support and family functionality.

## Introduction

Chronological age is a social category that involves inferences about people’s social and cognitive skills, making assumptions about the way they think and behave in relation to others (1–6).

Aging is a complex process in which factors such as culture, ethnicity, gender, health, physical and psychological well-being, social environment, work, education, and income determine the way in which people grow older (7, 8).

Maintaining and strengthening physical and cognitive functions are important for the process of active aging, as is an engagement in daily life, productive activities, and interpersonal relationships (8, 9). In this context, the various determinants of social support operate as mediators between people and the environment, affecting behavior, welfare, and health status (8, 10–13).

Society tends to venerate youth, caricaturing the process of aging as a condition involving shame and disgust (3, 14); this treatment causes elderly to experience age discrimination or ageism. Moreover, the perceived discrimination is significantly related to a change in depressive symptoms (15).

The concept of ageism can be applied to any age group (16, 17). Thus, the word *gerontoism*, a neologism adopted here, is applied only to the elderly and a form of discrimination mainly sustained by the maintenance of social stereotypes regarding age (5, 18, 19) and can occur in rational, emotional, and behavioral contexts (20).

On the assumption that ageism may result in damage to the welfare of the elderly, the aim of this study was to discuss, in a Brazilian context, if social support (SS) and family functionality (FF) can affect the self-perception of gerontoism (SG).

## Methods

This cross-sectional study sampled participants from Group Living centers in nine administrative regions, in Curitiba, Paraná, Brazil. This service is supported by the Foundation for Social Action (*Fundação de Apoio Social*, or FAS) and aims to facilitate the development of many activities to the elderly in a community setting.

In 2012 we selected 376 elderly from a population of 4325 people from the Group Living. A probabilistic sample was calculated, with *P*<0.05, and assuming a percentage of cases with positive SG of 50%. The participants should be 60 yr or older, age strata classified as elderly in Brazil (21). People with physical dependence and/or disabling mental conditions were not included in the sample.

The sociodemographic information collected was: birthplace, sex, age, skin color, marital status, existence of children, schooling, family income in minimum wages, and occupation.

To measure SS, we used the Medical Outcomes Study (MOS) scale (22) in a Brazilian version (23). This tool consists of 19 items distributed on five dimensions of SS: material = 4; affective = 3; emotional=4 items, information=4; positive social interaction= 4. The beginning of each item is worded, “If you need it, how often can you count on someone for…” Respondents select a value on an ordinal 5-point scale: 1 (never), 2 (rarely), 3 (occasionally), 4= (often), and 5= (always). Possible scores range from 3 to 15 for the dimension of effective support, and from 4 to 20 for the remaining dimensions. Total scores from the 5 dimensions were divided by the maximum of possible scores per dimension (3) and multiplied by 100 in order to standardize the scores. Final scores ranged from 20 to 100. Higher scores indicated greater individual experience of SS. For statistical analysis, the variable SS was used in its continuous form and was also dichotomized, with a median of 92 as the cut-off value, generating: high SS: ≥ 92; and low SS: < 92.

The FF was evaluated by the Family APGAR index (24), for Brazilian samples (25). This tool aimed to identify signs of family dysfunction (FD). The five basic units of FF are adaptability, partnership, growth, affection, and resolve. For each one, respondents could choose a value from an ordinal 3-point scale: 0 (hardly ever), 1 (some of the time), and 2 (almost always). Final scores ranged from 0 to 10. For the statistical analysis, FF scores were used as a continuous variable, as well as categorized as follows: high FD = 0–4 points, moderate FD = 5–6 points, and preserved FD = 7–10 points. The higher the score, the more is the individual perception related to the FF.

To assess the SG we used a version of the Ageism Survey (5, 18) and adjusted for Brazilian samples (26). It is composed of 20 questions, measuring the frequency of discriminatory situations suffered by respondents, and the experienced stress intensity (SI) associated with each situation. Responses regarding the frequency of occurrences (FO) were arranged on an ordinal 4-point scale: 0 (not applicable), 1 (never), 2 (sometimes), and 3 (often). The SI attributed to each event was arranged on an ordinal 3-point scale: 0 (no stress), 1 (moderate stress), 2 (high stress). The values of FO and SI were multiplied for each question. Mean scores were calculated and ranged from 0 to 6. Higher SG scores indicated more negative individual perceptions relating to ageism. We also included the option “dentist” with Question 12, which inquires whether a physician or nurse has supposed that the pain felt by the person was caused by old age. Similarly, for Question 13, which deals with the negative impact of age on medical treatment, we included the option “negative impact of age on dental treatment.” The response option “not applicable” was available only for Questions 6, 7, 8, 14, and 15, respectively, Q6, Q7, Q8, Q14, and Q15 of the scale.

The measures were administered during personal interviews. The interviews were carried out during Group Living meetings.

Data analyses were carried out using SPSS ver. 20.0 (SPSS IBM, New York, USA). Correlations between the continuous data of SG, SS, and FF were calculated using Pearson’s correlation coefficient. Comparisons between mean values of SG with the dichotomous values of SS and the sociodemographic variables were obtained using Student’s t-test for independent samples. Medium values of SG were compared with the categorical variables of FF and polytomous categorical sociodemographic variables using a one-way ANOVA and Tukey’s HSD test. The five dimensions of the MOS were compared with each other, and with the total scale score, using Pearson’s correlation coefficient; the aim was to identify the contribution of each dimension to the composition of the total score. In the same way, the five MOS dimensions were correlated with the total score of the Family APGAR index. Finally, the relationships between the MOS scores, the Family APGAR index scores, and scores on the Ageism Survey were examined using linear regression analysis.

The study was approved by the Research Ethics Committee of Pontifical Catholic University of Paraná, n.° 005112/11, and had technical approval from the FAS. All participants signed a statement of informed consent.

## Results

Table 1 shows the sociodemographic profile of the sample.

**Table 1:** Sociodemographic profile of elderly sample studied

***Sociodemographic variables***	***n***	***%***
***Birthplace***		
Capital	83	22.1
Metropolitan region	23	6.1
Small city from the interior	183	48.6
Midsize city from the interior	60	16.0
Large city from the interior	27	7.2
*Sex*		
Male	21	5.6
Female	355	94.4
*Age*		
60–69	171	45.5
70–79	158	42.0
≥ 80	47	12.5
*Skin color*		
White	286	76.1
Black	17	4.5
Brown	68	18.1
Yellow	4	1.1
Indigenous	1	0.2
*Marital status*		
Married/Cohabiting	135	35.9
Single	24	6.4
Widowed	177	47.1
Divorced/Separated	40	10.6
*Existence of children*		
Yes	353	93.9
No	23	6.1
Schooling^*^		
Never went to school	30	8.0
1^st^–4^th^ series	208	55.3
5^th^–8^th^ series	49	13.0
1°–2° high school	26	6.9
Complete high school	27	7.2
Superior and/or Post-graduate	36	9.6
Family income in minimum wage^*^		
≤ 1	91	24.2
1.1–2	118	31.4
2.1–5	100	26.6
> 5	29	7.7
Did not know or refused to say	38	10.1
*Occupation*		
Works and is not a retiree	21	5.6
Works and is a retiree	29	7.7
Householder	52	13.8
Unemployed	4	1.1
Pensioner	85	22.6
No activity and unpaid	9	2.4
Retiree and does not work	166	44.1
Beneficiary of continued provision	10	2.7

* These variables follow Brazilian criteria

Variables were: participants from inner cities (48.7%), female (94.4%), 60–69 yr (45.5%), white (76.1%), widowed (47.1%), with children (93.9%), with low schooling (55.3%), living with family income between one and two times the minimum wage (31.4%), and retirees not working (44.1%).

### Self-perception of gerontoism: frequency of occurrence and stress intensity

Table 2 shows the descriptive statistics of SG, FO, and SI.

**Table 2:** Descriptive statistics of self-perception of gerontoism

***Scores***	***Minimum***	***Maximum***	***Mean***	***Median***	***Standard Deviation***
SG	0.00	2.20	0.15	0.04	0.27
FO	0.75	2.00	1.01	0.95	0.24
SI	0.00	1.10	0.11	0.05	0.17

Note: SG – self-perception of gerontoism; FO – frequency of occurrences; SI - stress intensity

Values of SG ranged from 0.00 to 2.20, with a mean of 0.15. The maximum value that can be assumed in SG is 6, this indicates a low frequency of noted discriminatory situations. Furthermore, most of the participants categorized these situations as not stressful. The participants’ SG was therefore low.

Table 3 shows the ranking of questions according to the FO in situations described in the Ageism Survey and the SI attributed to each one.

**Table 3:** Decreasing ranking based on the frequency of occurrences of discriminatory events and attributed stress intensity (SI)

***Question***	***Text***	F^*^ (%)	***SI (%)***
1	Told me a joke that mocked older people	39.6	15.6
5	Spoke to me in a condescending or patronizing way because of my age	38.0	5.6
16	Suggested that I do not hear well because of my age	24.8	12.5
12	A physician, nurse, and/or dentist assumed that my pains are due to my age	24.2	15.1
17	Suggested that I do not quite understand because of my age	22.4	13.3
4	Called me a misnomer that insulted me due to my age	21.8	14.6
3	Was ignored or not taken seriously because of my age	21.3	17.1
18	Someone told me: “You are too old”	15.2	9.6
10	I was treated with less dignity and respect due to my age	13.6	11.5
20	I was a victim of violence (physical/moral) because of my age	8.8	8.7
19	My house was invaded because of my age	5.0	5.1
9	I was rejected for not being attractive because of my age	4.0	3.2
2	Sent me a birthday card that ridiculed older people	3.2	1.9
13	Denied me medical and/or dental treatment because of my age	3.2	2.7
7	I had trouble getting a loan because of my age	2.2	0.8
14	Denied me employment because of age	1.3	1.3
15	Denied me a promotion because of my age	1.3	1.1
6	Refused to rent me a house due to my age	1.0	0.6
11	A waiter ignored me because of my age	1.0	1.1
8	Denied me a management position because of my age	0.8	0.3

* Percentage of frequency of occurrences - excluding the options “not apply” and “never”.

The events most commonly perceived as discriminatory by the elderly were jokes that ridiculed them (Q1) and being treated in a condescending or patronizing way (Q5). However, Q1 showed a high value of attributed stress, while in Q5 the stress score was low.

### Social support and family functionality

The participants reported a high degree of SS, with a mean value of MOS of 87.5, with a possible range of 20–100.

Pearson’s correlation analysis showed that the dimensions of emotional support (*r* =0.81) and information support (*r* =0.80) were the most important in the construction of total MOS scores, followed by the dimension of social interaction support (*r* =0.74), effective support (*r* =0.73), and material support (*r* =0.72).

Examining the dichotomized scores for SS on the MOS, 48.9% of participants were classed as reporting high SS, with 51.1% reporting low SS.

Regarding FF, the medium value obtained by the Family APGAR index was 8.82. The categorical data for this measure showed that 87.5% of respondents indicated preserved FD, 7.7% reported moderate FD, and 4.8%, high FD. Pearson’s correlation revealed that the dimensions of affection, partnership, and adaptability (*r* = 0.81) influenced considerably the total scores on this scale, followed by development (*r* = 0.73) and resolve (*r* = 0.72).

### Self-perception of gerontoism according to social support, family functionality, and sociodemographic variables

Correlation analysis revealed a negative, weak, but statistically significant correlation between SG, SS, and FD (Table 4). Higher SG scores were related to lower scores of SS and FF.

**Table 4:** Pearson’s correlation results for variables self-perception of gerontoism (SG), social support (SS), and family functionality (FF)

***Variables***	***SG***	***SS***	***FF***
SG	1	−0.26	−0.28
SS	−0.26	1	0.53
FF	−0.28	0.53	1

Note: Bold values are statistically significant, *P* < .001

The Student’s t-test showed that participants who reported high levels of SS scored higher on SG (0.15 ± 0.19) than those who reported low levels of SS (0.20 ± 0.33), and this difference was statistically significant (*P* <0.001).

The one-way ANOVA revealed significant differences in SG scores between high, moderate, and low FD groups. The mean SG score was lower in the low FD group (0.12) than in the moderate (0.32) and high FD group (0.34). Tukey’s HSD test showed that a significant difference between the SG scores for respondents reporting low FD compared to the moderate dysfunction and high dysfunction groups (*P*=0.001 and *P*=0.003, respectively). However, there was no significant difference in SG means for the moderate and high FF groups (*P*=0.967).

There were no significant differences in SG scores on any of the sociodemographic variables (*P*>0.05).

The linear regression showed that scores on the five dimensions of the SS (MOS) scale explained 6.9% of the variance in SG (*R^2^* = 0.069). The variable of FF explained 7.8% of the variance in SG (*R^2^* = 0.078). Perceptions of SS and FF are not related to SG. The statistical analysis of correlation among the means of sociodemographic variables and SG showed no need for regression analysis.

## Discussion

Aging is a complex and multifaceted process, and to learn more about it and the way of life of the elderly can interfere with the social perspective (3, 14, 17, 26).

This study verified the existence of gerontoism in a Brazilian context (26). Many discriminatory events were experienced in the sample (17, 18, 26). In terms of those questionnaire items focusing on experiences such as trying to rent a house, look for a job, or a leading position, the low response rate revealed that these facts are not commonplace for this sample. A similar pattern was found before (17, 26).

This study evaluated the stress levels of participants associated with the discriminatory episodes experienced. Most respondents considered the episodes not stressful, a situation also observed (26). However, this does not mean that elderly do not notice particular discriminatory events. They do not consider such events stressful, or that they use protective strategies to cope with negative situations, such as instances of prejudice (26). In our study, this was evident with Q5. A high percentage reported that this had happened to them, but they had not perceived it as stressful.

In Brazilian culture, a more patronizing or condescending attitude with the elderly is often seen as an expression of care and intimacy, and not of disdain, contributed to the low reporting of stress in our sample.

The inclusion of an indicator of the level of stress clarified the extent to which the events of age discrimination impacted respondents’ welfare. Although discrimination may not be perceived as stressful by elderly, its occurrence remains a problem, particularly if they assume a discriminatory social label as normal because of their age (4, 20).

Age discrimination can be obscure, subtle, and difficult to decipher (20). One of the most elusive aspects of gerontoism is that it occurs unconsciously, implicitly and without intent to harm the target (27).

SS can be perceived as the extent to which interpersonal relationships respond to needs according to an individual’s level of satisfaction (28). In this study, we explored whether SG was related to a lack of SS. Gerontoism is lower in people with higher SS, while age discrimination is more prevalent in elderly who do not have much social protection. Thus, a lack of social relationships and support predispose people to gerontoism. These findings corroborate the observations by WHO that inadequate SS contributes to increased psychological problems and a decreased sense of overall well-being (7).

The family is a source of informal support and social interaction, and the relations of support among it influence the quality of aging (7). This natural group develops patterns of relationship that guide the internal operation of the family system and delineate responsibilities and behaviors of each of its members; in itself, it can be perceived as a resource or as a stressor (10, 29).

There are functional families that harmonize their relations in an integrated, functional, and effective way. However, there are dysfunctional families, which give priority to individual interests, where the emotional links are superficial and unstable and the family unit does not satisfactorily meet the needs of its members (11). The family promotes intergenerational interaction, and integration between generations is seen as a way to combat prejudice against the elderly (8). We found an inverse relationship between FF and SG.

Social integration is the key to elderly keep informed (8). In our study, for elderly, the most important affective relations occur at the family level. Australian elderly population were investigated formal support plays an important role in complementing informal support, and that both help to preserve the quality of life of elderly facing the decline of physical health (30). Close and supportive personal relationships are sources of emotional strength (7). The WHO highlights the importance of participation in leisure, cultural, and spiritual activities, both in society and in the family, allowing elderly to exercise their autonomy and enjoy respect and esteem from others, and stimulating the formation of supportive relationships and care (8).

We examined the socioeconomic profile of our sample, which showed similar patterns to those reported for this by IBGE (21). However, we did not find any relationship between sociodemographic variables and self-perception of gerontoism in our sample. Similar results were reported that sex, age, and schooling did not relate to scores on the Ageism Survey, but that socioeconomic level was a significant determinant of gerontoism (26).

Ageism and its determinants are noteworthy areas for future research. The increasingly aging global population makes this form of discrimination a compulsory subject on the agenda of all those interested in promoting a wide citizenship and a just society for all ages (4, 14, 20).

The elderly in our sample were not institutionalized, which could be able to actively maintain their social relations, while with the institutionalized the decline in physical and mental health, the loss of functional capabilities and the weakening of family and social ties are significant barriers to active aging (31). This fact may have contributed to the low overall mean scores found for the measure of SG. The sampling of a heterogeneous population in a specific geographical area can restrict the generalization of results. Our findings would suggest that future research involving vulnerable elderly without social relations might well find greater perceptions of gerontoism.

The version of the Ageism Survey used here has not been validated. However, it has undergone a linguistic adaptation (26) with the aim of making it more suitable for a Brazilian sample. On this scale, the option “not applicable” was adopted only for Q6, Q7, Q8, Q14, and Q15; participants were not given the option of choosing this response for other questions, perhaps obscuring the extent to which this scale was relevant to this sample. In addition, the response options “not applicable” and “never” for the frequency of occurrences were recorded by assigning a value of zero to the stress intensity. The two responses were not differentiated in the final scores on the scale.

## Conclusion

When elderly experience family conditions of increased functionality, and greater social support, they do not feel so victimized by age discrimination and are more likely to experience a pleasant old age.

## Ethical considerations

Ethical issues (Including plagiarism, informed consent, misconduct, data fabrication and/or falsification, double publication and/or submission, redundancy, etc.) have been completely observed by the authors.
